# *Helicobacter pullorum* in Chickens, Belgium

**DOI:** 10.3201/eid1202.050847

**Published:** 2006-02

**Authors:** Liesbeth M. Ceelen, Annemie Decostere, Kathleen Van den Bulck, Stephen L.W. On, Margo Baele, Richard Ducatelle, Freddy Haesebrouck

**Affiliations:** * Ghent University, Merelbeke, Belgium;; † Institute of Environmental Science and Research, Christchurch, New Zealand

**Keywords:** *Helicobacter pullorum*, poultry, isolation, PCR, amplified fragment length polymorphism, research

## Abstract

A total of 110 broilers from 11 flocks were tested for *Helicobacter pullorum* by polymerase chain reaction; positive samples were reexamined with a conventional isolation method. *H. pullorum* isolates were examined by amplified fragment length polymorphism (AFLP) fingerprinting for interstrain genetic diversity and relatedness. Sixteen isolates from cecal samples from 2 different flocks were obtained. AFLP analysis showed that these isolates and 4 additional isolates from a different flock clustered according to their origin, which indicates that *H. pullorum* colonization may occur with a single strain that disseminates throughout the flock. Strains isolated from different hosts or geographic sources displayed a distinctive pattern. *H. pullorum* is present in approximately one third of live chickens in Belgium and may represent a risk to human health.

## Abstract 2

*Helicobacter pullorum* is present in approximately one third of the chickens in Belgium.

*Helicobacter pullorum* was originally isolated from the feces and damaged livers of broilers and laying hens (*1**,**2*). It was defined as a new species in 1994 by Stanley et al. (*1*). *H. pullorum* is a gram-negative, slightly curved rod with monopolar, nonsheathed flagella. It is bile resistant and requires a microaerobic environment supplemented with H_2_ in which growth occurs at 37°C and 42°C (*1**,**3**–**6*). Enterohepatic *Helicobacter* species, including *H. pullorum*, are increasingly recognized as microbial pathogens in humans and animals (*3**,**5**,**7**–**9*). *H. pullorum* has been linked with enteritis and hepatitis in broiler chickens and laying hens and diarrhea, gastroenteritis, and liver disease in humans (*1**,**2**,**5**–**8**,**10**,**11*). *H. pullorum* can contaminate poultry carcasses at the abattoir and can be considered a foodborne human pathogen (*4**,**8**,**12*).

Almost no data are available on the prevalence of this species in poultry. Research that could generate these data is hampered by the fastidious growth requirements of *H. pullorum* and the phenotypic similarity between member species of the genera *Helicobacter* and *Campylobacter* (*3**,**4**,**12*). *H. pullorum* in chickens has been studied on only 2 occasions when the organism was detected by using isolation (*4**,**7*). Furthermore, no valid epidemiologic research methods have been recommended.

This study's objective was to determine the occurrence of *H. pullorum* in broilers by using both polymerase chain reaction (PCR) and isolation. In addition, amplified fragment length polymorphism profiling (AFLP) was conducted to investigate the genetic relatedness between *H. pullorum* isolates.

## Methods

### Sample Origin

Samples from the gastrointestinal tracts and livers of 110 broiler chickens, 10 per flock (flock number 1–11), collected at a poultry abattoir, were studied. Each gastrointestinal tract and liver sample was deposited in a separate waterproof plastic bag. Samples were taken from the liver, cecum, jejunum, and colon for PCR and isolation within 3 hours after collection. All samples were stored at –20°C and –70°C for PCR and isolation, respectively, until further analysis, as described below.

### Sample Processing

#### PCR and Gel Electrophoresis

DNA was extracted from ≈25 mg cecum, colon, jejunum, and liver tissue with a commercial tissue kit (DNeasy Tissue Kit, Qiagen, Venlo, the Netherlands). A PCR assay amplifying a 447-bp fragment of the 16S rRNA gene of *H. pullorum* was then used for detection purposes (*1*). From each sample, 2 μL template was added to 8 μL PCR mixture containing 0.03 U/μL Taq polymerase Platinum (Invitrogen Life Technologies, Merelbeke, Belgium), 10× PCR Buffer (Invitrogen Life Technologies), 3 mmol MgCl_2_ (Invitrogen, Life Technologies), 40 μmol/L each of deoxynucleoside triphosphate (Invitrogen Life Technologies), a final primer concentration of 0.5 μmol/L, and sterile distilled water. The conditions used for the amplifications were the following: an initial denaturation at 94°C for 5 min, followed by 35 cycles of denaturation at 94°C for 1 min, annealing at 60°C for 90 s, elongation at 72°C for 90 s, and a final elongation at 72°C for 5 min.

Five microliters of the PCR products of each sample were mixed with 3 μL of sample buffer 5× (50% glycerol, 1 mmol cresol red) and were subjected to electrophoresis through an agarose gel containing 1.5% Multi Purpose agarose (Boehringer, Mannheim, Germany) and 50 ng ethidium bromide in per milliliter 1× Tris-acetate ethylenediaminetetraacetic acid buffer (Amresco, Solon, OH, USA), pH 8. As molecular size marker, the Gene Ruler 100-bp DNA ladder plus (MBI Fermentas, St. Leon-Rot, Germany) was used. Electrophoresis was implemented at a constant voltage of 170 V in 0.5× Tris-acetate ethylenediaminetetraacetic buffer for 75 min. The gels were visualized by using the Image Master VDS (Pharmacia Biotech, Puurs, Belgium).

#### Isolation of *H. pullorum*

Recovery of *H. pullorum* isolates was attempted on all positive samples in the PCR analysis described above. The samples (200 mg) for isolation of *H. pullorum* were placed in a 1.5-mL tube with 400 μL of a mixture of 7.5 g glucose, 25 mL brain heart infusion broth (Oxoid, Basingstoke, England), and 75 mL sterile inactivated horse serum, and then homogenized. The various isolates were inoculated on brain heart infusion agar that was supplemented with 10% horse blood, amphotericin B 20 μg/mL (Fungizone, Bristol-Myers Squibb, Epernon, France), and Vitox (Oxoid) (blood agar). A modified filter technique of Steele and McDermott (*13*) was then used. Briefly, a sterile cellulose acetate membrane filter (0.45 μm) was applied with a sterile pair of tweezers directly onto the surface of the agar. When the filter was totally absorbed on the agar, ≈300 μL of the mixture was placed in the middle of the filter. After at least 1 hour of incubation at 37°C and 5% CO_2_, the filter was removed with a sterile pair of tweezers and the filtrate was streaked on the agar with a loop. Incubation was conducted in microaerobic conditions (5% H_2_, 5% CO_2_, 5% O_2_, and 85% N_2_) at 37°C for a minimum of 3 days. Very small, gray-white, hemolytic colonies were selected and purified on a blood agar plate. The colonial form and phenotypic characteristics (gram-negative, slightly curved rod, catalase and oxidase positive, and indoxyl acetate negative) of the isolates were used for presumptive identification. Confirmation was based on PCR and sequencing of a 447-bp fragment of the 16S ribosomal RNA gene, as described below.

Analysis of Nucleotide Sequences

The PCR product of the retrieved *H. pullorum* isolates was purified with the Qiaquick purification kit (Qiagen) and sequenced by using the same primers applied in the assay with the BigDye Terminator cycle sequencing kit (Applied Biosystems, Lennik, Belgium). Sequencing products were run on the ABI prism 3100 Genetic Analyzer (Applied Biosystems) by using 50-cm capillaries filled with Performance-Optimized-Polymer 6. The electrophoregrams were exported and converted to the Kodon software package (Applied Maths, Sint-Martems-Latem, Belgium). Sequences were compared to published *H. pullorum* 16S rRNA sequences obtained from GenBank (accession nos. AY631956, L36143, and L36144) by using BLAST software (available from http://www.ncbi.nlm.nih.gov/blast/).

### AFLP

Twenty-two poultry and 3 human isolates were fingerprinted by using AFLP (Table 1). These included 16 isolates from flock numbers 5 and 9 screened in this study. In addition, 4 samples previously isolated from broilers' cecal droppings and the boots from another flock's farmer, 4 reference strains (2 of chicken and 2 of human), and 1 human strain isolated from diarrheic stool in our laboratory were included for comparison.

**Table 1:** *Helicobacter pullorum* isolates studied by using AFLP*

Strain	Flock	Source	Geographic origin
CE III 2	Flock CLO	Cecal droppings, broiler chicken	Belgium
CE III 3		Cecal droppings, broiler chicken	
CE III 4		Cecal droppings, broiler chicken	
CE III 5		Worker's boot	
CE II 1	Flock no. 5	Cecal tissue, broiler chicken	Belgium
CE II 2		Cecal tissue, broiler chicken	
CE II 3		Cecal tissue, broiler chicken	
CE II 4		Cecal tissue, broiler chicken	
CE II 5		Cecal tissue, broiler chicken	
CE II 6		Cecal tissue, broiler chicken	
CE II 7		Cecal tissue, broiler chicken	
CE II 8		Cecal tissue, broiler chicken	
CE I 1	Flock no. 9	Cecal tissue, broiler chicken	Belgium
CE I 2		Cecal tissue, broiler chicken	
CE I 3		Cecal tissue, broiler chicken	
CE I 4		Cecal tissue, broiler chicken	
CE I 5		Cecal tissue, broiler chicken	
CE I 6		Cecal tissue, broiler chicken	
CE I 7		Cecal tissue, broiler chicken	
CE I 8		Cecal tissue, broiler chicken	
CCUG 33837	NA	Healthy broiler chicken	Switzerland
CCUG 33840	NA	Laying hen, hepatitis	Switzerland
CCUG 33838	NA	Stool, gastroenteritis and hepatitis, human	Switzerland
CCUG 33839	NA	Stool, gastroenteritis, human	Switzerland
G 214	NA	Stool, gastroenteritis, human	Belgium (*11*)

*AFLP, amplified fragment length polymorphism; CLO, Centrum voor Landbouwkundig Onderzoek; CCUG, Culture Collection of the University of Göteborg; NA, not applicable or available

#### Restriction Endonuclease Digestion and Ligation of Adaptors for AFLP

DNA of *H. pullorum* isolates was extracted by using a commercial tissue kit (DNeasy Tissue Kit, Qiagen). An aliquot containing 200 ng DNA, determined by optic density (260/280 nm) measurement by using the Spectra Fluor (TECAN, Grödig, Salzburg, Austria), was digested for 2 h at 37°C with *Bgl*II (10U/μL) and *Csp*6I (10U/μL) (MBI Fermentas) in TAC-buffer as described by Vos et al. (*14*). Five microliters of DNA digest was used in a ligation reaction containing 130 μg/mL *Bgl*II adaptor-oligonucleotide and 13 μg/mL *Csp*6I adaptor-oligonucleotide (Invitrogen) (*14*), 10× T4 DNA ligase buffer, T4 DNA ligase (1 U/μL) (Amersham Pharmacia), and TAC-buffer in a final volume of 20 μL. After incubation for 2 h at 25°C, the 20 μL ligation reaction was diluted 25 times.

#### Direct Selective PCR Amplification of Diluted Ligation

Five microliters of the diluted ligation reaction were applied in the PCR assay. The primers used in this assay were BGL2F-0, 5´-GAG TAC ACT GTC GAT CT-3´ (FAM labeled, 5´-end) and CSP6I-A, 5´-GAG CTC TCC AGT ACT ACA-3´ (*15*). The PCR conditions were as follows: an initial denaturation at 94°C for 3 min; 35 cycles of denaturation at 94°C for 1 min, annealing at 54°C for 1 min, and elongation at 72°C for 90 s; and a final elongation at 72°C for 10 min.

#### Capillary Electrophoresis

PCR products were run on the ABI prism 3100 Genetic Analyzer (Applied Biosystems) by using the Fragile X Rox-1000 size standard and 50-cm capillaries filled with Performance-Optimized-Polymer 6. Electropherograms were analyzed with Genemapper U 3.5 Software (Applied Biosystems).

#### Numerical Analyses of AFLP Profiles

The program BioNumerics version 2.5 (Applied Maths) was used to perform numerical analyses of AFLP profiles. Strain relationships were inferred by use of the Pearson product-moment correlation coefficient and unweighted pair-group with mathematical average (UPGMA) clustering and depicted in a dendrogram (*16*).

## Results

### PCR

In Table 2, the number of *H. pullorum* DNA–positive samples originating from the intestinal tract and liver is shown. In 4 flocks, all samples were negative for *H. pullorum*. In the other 7 flocks, positive samples were found. In the cecum and colon, a PCR reaction for *H. pullorum* was positive in 33.6% and 31.8% of the samples, respectively. In total, 10.9% of jejunum and 4.6% of liver samples were positive for *H. pullorum*.

**Table 2:** No. *Helicobacter pullorum*–positive poultry tissue samples by polymerase chain reaction

Flock no.	No. positive samples*			
	Cecum	Colon	Jejunum	Liver
1	2	2	1	0
2	3	8	4	0
3	4	1	1	0
4	7	4	1	0
5	8	8	0	0
6	0	0	0	0
7	4	4	0	1
8	0	0	0	0
9	9	8	5	4
10	0	0	0	0
11	0	0	0	0
Total	37	35	12	5

*No. positive animals of 10 screened per flock.

### Isolation of *H. pullorum*

Eight *H. pullorum* cecum isolates from flock number 5 and 8 *H. pullorum* cecum isolates from flock number 9 were obtained. The sequences of the amplified 447-bp fragment of the *H. pullorum* 16S ribosomal RNA gene isolates showed a similarity of 98%–100% to those from GenBank (accession nos. AY631956, L36142, and L36143).

### AFLP

AFLP analysis showed that isolates from each of the individual flocks examined clustered according to their flock of origin. The remaining chicken isolates and human strains each displayed a unique profile (Figure).

**Figure:**
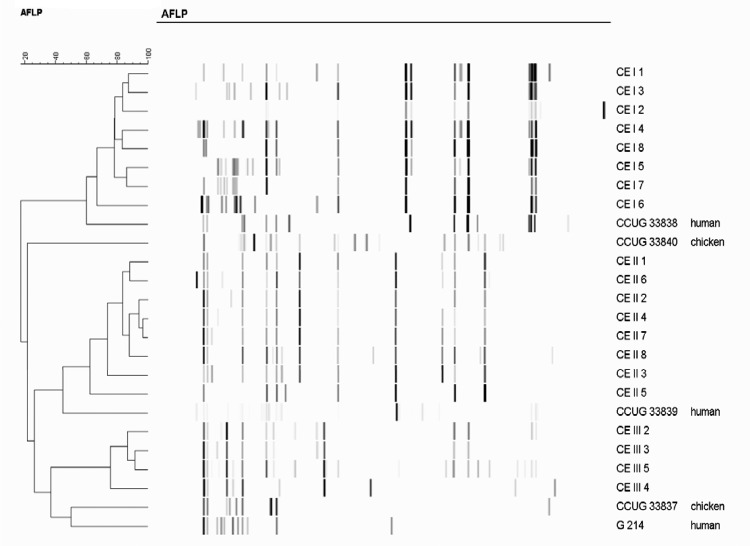
Chicken isolates and human strains of *Helicobacter pullorum* by amplified fragment length polymorphism.

## Conclusion

This study shows that *H. pullorum* is present in 33.6% of the cecal samples of broiler chickens collected at a poultry slaughterhouse during evisceration by using PCR. This microorganism was found in 7 of 11 flocks; 4 flocks were negative. Burnens et al. found a prevalence rate of 4% upon sampling cecal contents of broilers (*7*). The organism was detected by isolation. Considering the fastidious nature of this organism, this finding could explain this markedly lower percentage of positive birds. Additionally, in our study, cecal tissue, rather than cecal contents, was examined for the organism. Microorganisms related to *H. pullorum* adhere closely to the mucosa of the gastrointestinal tract. The phylogenetically related microorganism, *C. jejuni*, may tightly adhere to the brush borders of the intestine in chickens (*17**,**18*). The same phenomenon has also been documented for *H. pylori* in the stomach (*19*).

Comparing our study results to those obtained by Atabay et al. (*4*), the latter group found a higher occurrence of *H. pullorum* (60%) on poultry carcasses. This apparent discrepancy could be due to cross-contamination with cecal contents on the surface of broiler carcasses during poultry processing (*4**,**8*). Furthermore, contamination of the chicken body surface may occur during transportation to the abattoir. Fecal excretion of *Campylobacter* spp. may be increased because of stress during transportation and consequently may contaminate carcasses (*20*).

*H. pullorum* DNA was detected in only 5 (4.6%) liver and 11 (10.9%) jejunal samples, as opposed to 35 (31.8%) colonic and 37 (33.6%) cecal samples. Hence, one may assume that the lower segments of the intestinal tract are the predominant colonization sites for *H. pullorum* in broiler chickens. *H. pullorum* may gain access to the liver by retrograde transfer from the duodenum. Alternatively, it may translocate from the gut lumen to the portal circulation.

*H. pullorum* has been associated with vibrionic hepatitis in laying hens, both macroscopically and microscopically (*7*). In our study, no gross pathologic lesions were seen in the livers during sampling (data not shown).

Our modest isolation rate of *H. pullorum* from cecal samples may have been the result of examining frozen, as opposed to fresh, samples. However, we successfully recovered 16 isolates from 2 flocks, allowing (for the first time, to our knowledge) some analysis of the etiology of *H. pullorum* in broiler flocks to be undertaken. We used AFLP profiling for this purpose, a highly discriminatory method that has been successfully applied to molecular epidemiologic studies of several related species, including *H. pylori* (*21**,**22*), *Arcobacter* spp (*15*), and *Campylobacter* spp (*23**,**24*). Isolates from each of the individual flocks clustered according to their flock of origin, indicating a clonal relationship. In contrast, field and reference strains isolated from different hosts or geographic sources displayed a distinctive pattern. These data suggest that AFLP profiling has considerable potential for molecular epidemiologic studies of *H. pullorum* for the noted related species.

Several authors have suggested that *H. pullorum* has zoonotic potential and is involved in the pathogenesis of diarrhea and chronic liver diseases in humans (*2**,**8**,**10**,**11*). Retail raw poultry meats and other poultry products may constitute vehicles for human *H. pullorum* infections through carcass contamination, as previously reported for *Arcobacter* and *Campylobacter* species (*8**,**25**–**27*). Concerning health monitoring, PCR may be helpful in detecting this pathogen not only in intestinal tissue but also in broiler chicken cecal droppings.

In conclusion, this study shows that *H. pullorum* is a frequent intestinal colonizer of broiler chickens. PCR and isolation are useful tools to detect the species in intestinal tissue and in cecal droppings. AFLP profiling appears to be useful for molecular epidemiologic studies of this species.
